# Regime shifts in coastal lagoons: Evidence from free-living marine nematodes

**DOI:** 10.1371/journal.pone.0172366

**Published:** 2017-02-24

**Authors:** Sergio A. Netto, Gustavo Fonseca

**Affiliations:** 1 Marine Science Laboratory, University of Southern Santa Catarina, Tubarão, Santa Catarina, Brazil; 2 Departamento de Ciências do Mar, Universidade Federal de São Paulo, Santos, Brazil; Universita degli Studi di Genova, ITALY

## Abstract

We test the validity of using the regime shift theory to account for differences in environmental state of coastal lagoons as a response to variation in connectivity with the sea, using free-living nematodes as a surrogate. The study is based on sediment samples from the inner and outer portions of 15 coastal lagoons (5 open to the sea, 5 intermittently open/closed, and 5 permanently closed lakes) along the southern coast of Brazil. Environmental data suggested that there are two contrasting environmental conditions, with coastal lakes being significantly different from open and intermittent lagoons. Marine nematode assemblages corroborate these two mutually exclusive alternative stable states (open vs. closed systems), but assemblages from the intermittently open/closed lagoons showed a gradual change in species composition between both systems independently of the environmental conditions. The gradient in the structural connectivity among lagoons and the sea, due to their regime shifts, changes the movement of resources and consumers and the internal physico-chemical gradients, directly affecting regional species diversity. Whereas openness to the sea increased similarity in nematode assemblage composition among connected lagoons, isolation increased dissimilarity among closed lagoons. Our results from a large-scale sampling program indicated that as lagoons lose connectivity with the sea, shifting the environmental state, local processes within individual intermittently open/closed lagoons and particularly within coastal lakes become increasingly more important in structuring these communities. The main implication of these findings is that depending on the local stable state we may end up with alternative regional patterns of biodiversity.

## Introduction

**Fig 1:**
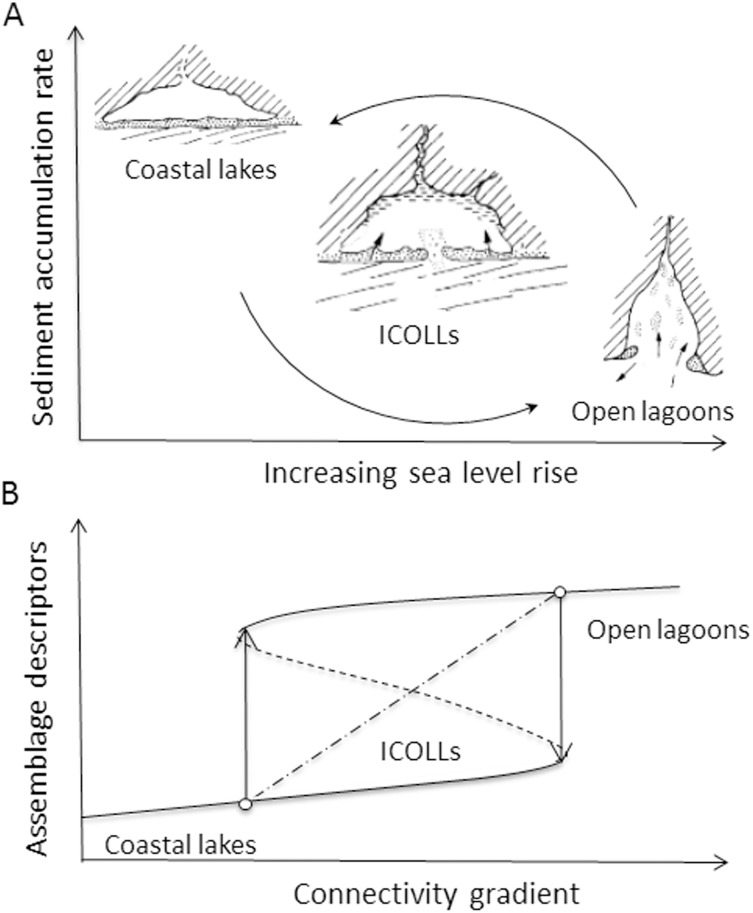
Conceptual model of coastal lagoon evolution based on the relative importance of sedimentation, river inflow and tide (A) and the model showing the transition between the two alternative stable states of coastal lagoons along a connectivity gradient (B).

Coastal lagoons are transitional aquatic systems that mediate transfers between the terrestrial environment and the ocean, including potential environmental stressors [1,2]. Lagoons are an evolving coastal landform that may go through a cycle from an open embayment, to a partially back-barrier lagoon with progressive infilling, to a segmentation into small lagoons with unstable inlets and then lakes [3,4] (Fig 1A). The evolution of coastal lagoons is the result of the balance between the processes which act to reduce the size of a lagoon and those, which act to increase it [5]. For a given lagoon status, the combination of rate of accretion and sea rise will determine the volumetric capacity of the lagoon, its import/export status, and the resultant evolution [6]. The relative importance of a particular process in a lagoon depends upon the environmental setting in which the lagoon is located and the evolutionary path followed by a lagoon depends upon the magnitude and relative importance of each of the operative processes [5]. The dynamism of these forces promotes both long-term and short-term changes in these ecosystems. In the long-term (months and years), it influences the connectivity with the sea, while in the short-term (tidal cycles), it affects the amount of seawater inflow. According to the present connectivity with the sea, these coastal water bodies can roughly be divided into three major types: open (permanently connected to the sea), the intermittently open/closed (which includes seasonally or non-seasonally closed or those normally closed), and the closed (presently without permanent open bar, the coastal lakes).

Open lagoons are characterized by a wide spatio/temporal range in environmental conditions (e.g., salinity, temperature, oxygen), biological productivity and movement of resources and consumers with other adjacent marine areas [7,8]. In contrast, coastal lakes are largely more homogeneous in their environmental conditions than open lagoons. Intermittently open/closed lagoons and lakes (ICOLL) show dramatic environmental changes over a short period of time, especially concerning hydrodynamics, salinity gradient, sediment composition and concentration of organic matter [9,10,11]. This shift from a completely open coastal lagoon to a coastal lake causes abrupt changes in the biota [12,13] as expected by the alternative stable state model [14] (Fig 1B dashed line). The environmental shift might be induced by natural processes over geological scales, or by anthropogenic activities at the ecological scale, such as hydrological management [15], artificially connecting coastal lakes to the sea [9] or modifications as a result of climate change [16].

Although the shift in biodiversity patterns is theoretically sound, there is a lack of empirical evidence to support it. So far, shifts in biodiversity patterns for coastal lagoons have been restricted to single lagoons and water column assemblages [12,13]. The benthic system has gained little attention. Typically, the benthic systems of open lagoons are composed of a number of estuarine resident and many temporary marine species [17,18,19]. The species composition in intermittent lagoons may be variable according to the current connectivity state. After blocking events (depending on rainfall regime and time of closure), they might become more homogeneous and dominated by freshwater species, typical of coastal lakes [9, 20,21,22]. These isolated observations suggest that the benthic system may not respond gradually after a blocking/opening event (dot-dashed line, Fig 1B), but may respond abruptly showing two alternative stable states (dashed line, Fig 1B).

In this study, we investigate to what extent the differences in openness of coastal lagoons structure meiofauna communities. Meiofauna comprises a group of benthic organisms ranging from 0.5 mm to 0.05 mm [23]. They are omnipresent in all types of marine habitats occurring in high abundances and number of species. Given their short life cycles and tight relationship with the sediment composition [23], meiofauna is an ideal tool to investigate short- to long-term changes in coastal lagoons. We assume that open and closed lagoons are two alternative states of equilibria, and that intermittent lagoons are the transition phase between them. Based on this assumption, we expect that the benthic system will respond accordingly showing two alternative stable states. Additionally, we expect that (1) open lagoons will have higher regional richness and abundance as resources and consumers move among adjacent habitats; (2) absence of barriers and fauna movements by outlets will increase similarity between open lagoons, while the isolation would increase dissimilarity (species turnover) between closed lagoons; (3) openness generates environmental gradients which will increase dissimilarities within lagoons.

## Materials and methods

### Coastal lagoon sampling and sample processing

**Fig 2:**
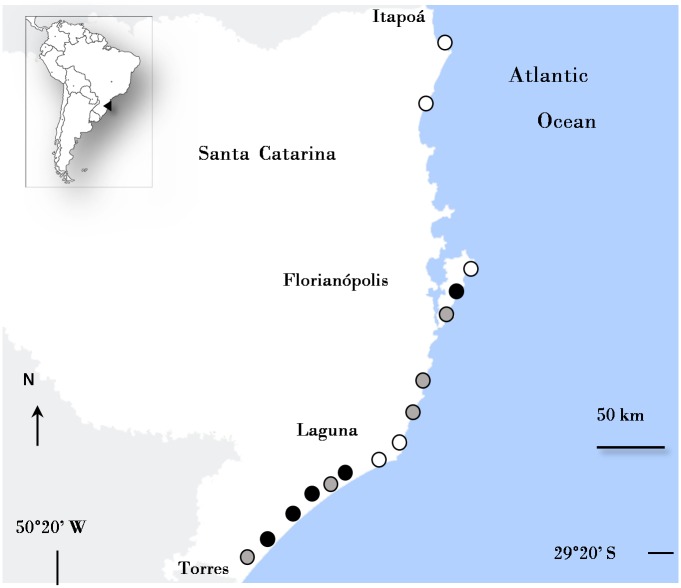
Map of the coast of Santa Catarina showing the location of the sampled lagoons: Closed (black circle), intermittently open/closed (gray circle) and open (open circle).

Coastal lagoons were sampled along the ~430 km coast of Santa Catarina State, South Brazil (Fig 2). This coastline can be divided into two major segments:—from the north border, in Itapoá, up to the Cape Santa Marta, at Laguna, the coast is N-S orientated, highly embayed with rocky headlands alternating with small bays;—southwards of Laguna, the straight NE-SW coast is dominated by high energy sandy shores. The most frequent swell wave direction along this coast is from the south, with average heights of 2.5 m; the coast has a micro-tidal regime with higher tides in the north (mean astronomic tide 1 m in Itapoá) than in the south (0.5 in Laguna); the general alongshore littoral drift is from S-SE to ENE-NE, but local reversals take place during strong NE conditions [24]. Coastal lagoons are mainly concentrated in central/southern portions of the coast (Fig 2).

Fifteen coastal lagoons/lakes (5 closed- Peri, Jaguaruna, Faxinal, Esteves, Cavera; 5 open—Camacho Laguna, Conceição, Barra Velha, São Francisco; 5 intermittent -Lagoinha do Leste, Garopaba, Ibiraquera, Urussanga, Sombrio) were sampled in the austral summer 2012. The lagoons are marginally urbanized, and sampling points have been selected out of the urbanization range to avoid potential influence of anthropogenic impacts. In each of the lagoons, 9 meiofauna samples and 3 of sediment samples (granulometry and total organic content) were taken in the outer and inner portions of the lagoons (a total of 18 samples of meiofauna and 6 samples of sediment per lagoon). The samples of each sample portion were taken dozens of meters apart from each other. For the closed lagoons, inner samples were those taken westward in the most interior region, while the outer samples were taken eastward near the sand barrier (exact location of sampling sites are available in S1 Table). Water salinity was measured in situ with a multiparameter YSI. Meiofaunal shallow subtidal samples (<1m depth) were taken with a plastic syringe of 2 cm in diameter pushed to a depth of 10 cm. They were immediately fixed in 4% formalin, later sieved through a 63 μm mesh and extracted by flotation with Ludox TM (specific gravity: 1.18). Samples were then evaporated with anhydrous glycerol and permanent slides were made [25]. Sediment samples were taken with a PVC corer tube (10 cm Ø and 5 cm height). Granulometry was done by sieving and pipetting analysis and total organic content was determined by loss of ignition (550°C for 4 hours). Carbonate content of sediments samples was determined by acid digestion [26].

### Data analysis

Nematode univariate descriptors were the number of genera (richness; S), density (inds. 10 cm^-2^; N) and Shannon-Wiener diversity index (log_2_; H'). As functional attributes of the assemblages across the studied lagoons, we analysed the nematode feeding types [27,28,29], and the index of trophic diversity (1-ITD) [30] based on the proportion of each feeding type. Because the study encompasses both marine/estuarine and freshwater nematodes we used five feeding types: selective deposit feeders (1A), nonselective deposit feeders (1B), epigrowth feeders (2A), predators/omnivores (2B) and vascular plant feeders (3).

Differences in nematode descriptors and functional attributes among typologies (fixed factor: closed, ICOLL and open), lagoon (random factor: 5 closed, 5 open and 5 ICOLL, nested in typology) and location (random factor: inner and outer, nested in lagoons and nested in typology) were tested with a permutational analysis of variance (PERMANOVA) run on Euclidean distance matrices with 9999 permutations, and the residuals were permuted under a reduced model [31]. To visualize the similarity of the meiofauna composition among different lagoon typologies and location within lagoons, similarity matrices were constructed based on the Bray-Curtis similarity measure. Ordination was done by nMDS, and significance tests for differences in the multivariate structure of nematode assemblages performed using PERMANOVA [31]. The variation in species composition of nematode assemblages (beta diversity) was decomposed into replacement and richness difference using abundance data dissimilarities and the Sorensen index [32]. These analyses were performed using the R software [33]. Total β-diversity and decomposed replacement and richness differences were analyzed using PERMANOVA tests with the same design described above. The decomposition of beta diversity can be done by two methods, the “POD” and “BAS” [32]. Although both indices may not show congruent patterns [32,34], in the present study they showed agreement for total dissimilarity and replacement. For richness, the BAS method returned negative sums of squares for the factor typology, while the POD did not (see Results).

Differences in the environmental variables (salinity, mean grain size, sorting, total organic content, sand, silt and clay percentages) were also tested using PERMANOVA using the same design as for the fauna. The relationships between environmental variables and nematode assemblages were explored using distance-based redundancy analysis (dbRDA) that enabled us visualize the percentage of variability in the original data explained by the axis and the relative contributions of each of the predictor variables on the assemblage structure [31].

### Ethic statement

No specific permits were required to collect meiofauna as they are microscopic, non-pathogenic and with no special conservation concerns. Field study did not involve endangered species and sampling was carried out in public waters.

## Results

### Faunal descriptors and connectivity

A total of 106 genera of nematodes was recorded, among which 19 were recorded in the closed lagoons, 71 in the intermittently open/closed, and 68 in the open lagoons (Table 1). Most of the nematode genera recorded in open lagoons (70.5%) were those typically found in brackish or marine waters. This proportion was reduced in ICOLLs (49%) with an increasing number of brackish/freshwater or freshwater genera. In closed lagoons, freshwater or brackish/freshwater genera accounted for 95% of the collected fauna. Only 5 genera occurred in all the three types of lagoons, namely *Anonchus* (Aphanolaimidae), *Anoplostoma* (Anoplostomatidae), *Desmodora* (Desmodoridae), *Dichromadora* and *Hypodontolaimus* (Chromadoridae). The percentage of exclusive genera (those found exclusively in only one type of lagoon) decreased with increasing connectivity: 13 genera occurred exclusively in the closed lagoons (65%), 26 in the intermittently open/closed (35%) and 21 in open lagoons (30%; Table 1). *Trischistoma* (Trypilidae), *Semitobrilus* (Trobilidae) and *Ironus* (Ironidae) were the most abundant genera in closed lagoons, accounting for 55% of the nematodes collected. At the intermittently open/closed and open, the genera *Microlaimus* (Microlaimidae), *Spirinia* and *Desmodora* (Desmodoridae) were the most abundant genera in both types of lagoons (Table 1).

**Table 1:** Summary characteristics of nematode assemblages from coastal lagoons.

	Closed	ICOLL	Open
Number of genera	20	73	69
Number of freshwater genera	6	14	1
Number of freshwater / brackish genera	12	22	19
Number of brackish genera	1	35	48
Exclusive genera (%)	65	35	30
Most frequent genera	*Semitobrilus* (60%) *Trischistoma* (48%)	*Desmodora* (81%) *Theristus* (80%)	*Desmodora* (78%) *Theristus* (77%)
Total density (inds.10 cm^-2^)	3–407 (57)	9–5474 (678)	6–5283 (674)
*Trischistoma* (inds.10 cm^-2^)	29	-	-
*Semitobrilus* (inds.10 cm^-2^)	21	-	-
*Ironus* (inds.10 cm^-2^)	5.2	0.07	-
*Microlaimus* (inds.10 cm^-2^)	-	143	80
*Spirinia* (inds.10 cm^-2^)	-	108	185
*Desmodora* (inds.10 cm^-2^)	0.04	73	37

Total number of genera, number of brackish/freshwater genera of according to [35,36], percentages genera found exclusively in lagoon types (exclusive genera), frequent genera, minimum–maximum densities of nematodes (and average inds.10cm^-2^), and the most abundant genera (inds.10cm^-2^) in closed, intermittently open/closed and open lagoons of Santa Catarina coast, South Brazil. A complete list of nematode genera, environment (brackish/freshwater), and mean densities in each lagoon typology can be found in S2 Table.

**Fig 3:**
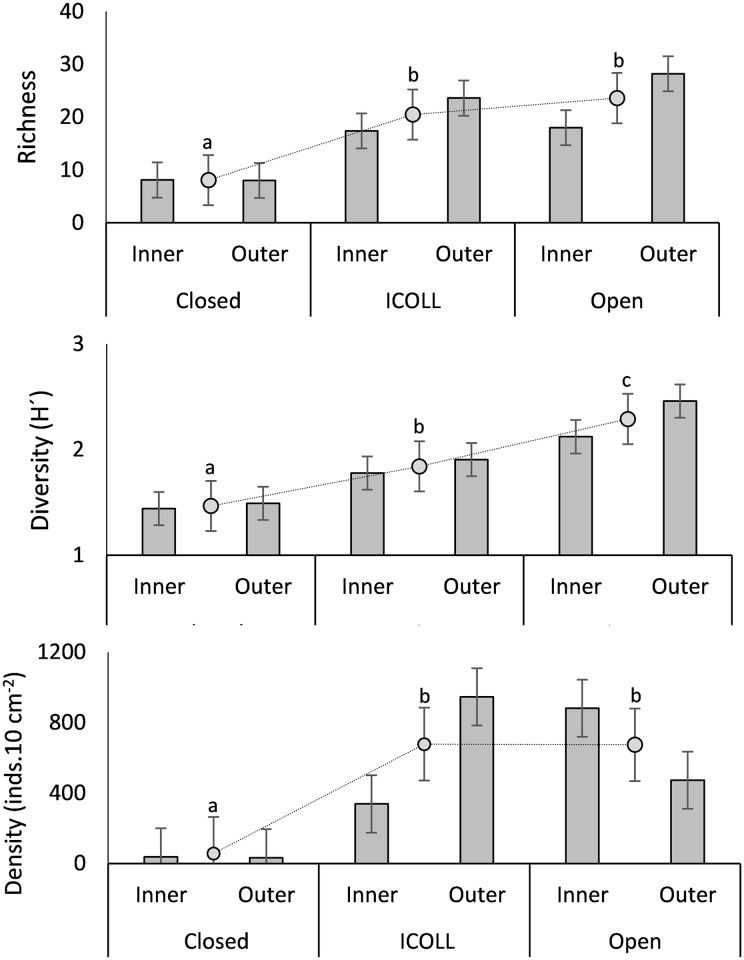
Mean (±SE) of nematode number of genera, diversity and density in inner and outer portions (columns) of lagoons, and total mean value (±SE) of the descriptor in closed, intermittently open/closed (ICOLL) and open lagoons (gray dot). Different letters indicate significant differences (p<0.05) among lagoon typology.

The number of genera and diversity of nematodes were significantly higher in the open lagoons, followed by intermittent and were lowest at closed ones (Table 2 and Fig 3). Density was significantly higher in open lagoons and ICOLLs than in closed ones (Fig 3). Differences in the univariate measures between individual lagoons/lakes occurred mostly within the closed ones (S3 Table). Significant differences between outer and inner portions increased with lagoon connectivity. In the closed lagoons, the descriptors did not show any significant differences between inner and outer portions; in ICOLLs, nematode richness, diversity and density were, in general, higher in outer portion, or did not differ significantly (S4 Table). All descriptors differed significantly in open lagoons, where richness and diversity were higher in the outer portion and density was higher in the inner parts of the lagoons (Fig 3 and S4 Table).

**Table 2:** Permutational analysis of variance (PERMANOVA) results testing the effects of lagoon typology (Open, ICCOL and Closed), lagoons (5 open, 5 ICOLL and 5 closed) and location (inner and outer) on the univariate nematode descriptors, feeding types and index of trophic diversity.

	Sources of variation	df	SS	MS	Pseudo-F	P(MC)
Richness	Typology	2	3035.4	1517.7	6.6401	**0.008**
	Lagoon (Typology)	12	2749.1	229.09	3.2699	**0.02**
	Location[Lagoon(Typology)]	15	1051.5	70.098	11.249	**0.001**
	Residual	235	1464.3	6.2313		
Shannon diversity	Typology	2	25.519	12.76	6.8144	**0.01**
	Lagoon (Typology)	12	22.518	1.8765	1.8651	0.131
	Location[Lagoon(Typology)]	15	15.099	1.0066	6.7646	**0.001**
	Residual	235	34.969	0.1488		
Density	Typology	2	235.8	117.9	5.0229	**0.034**
	Lagoon (Typology)	12	282.31	23.526	3.233	**0.021**
	Location[Lagoon(Typology)]	15	109.21	7.2807	11.872	**0.001**
	Residual	235	144.12	0.6132		
Index of trophic diversity	Typology	2	1.2518	0.6258	9.9346	**0.007**
	Lagoon (Typology)	12	0.7591	0.0632	0.6606	0.775
	Location[Lagoon(Typology)]	15	1.4382	0.0958	0.9482	0.235
	Residual	235	5.561	0.2428		

**Fig 4:**
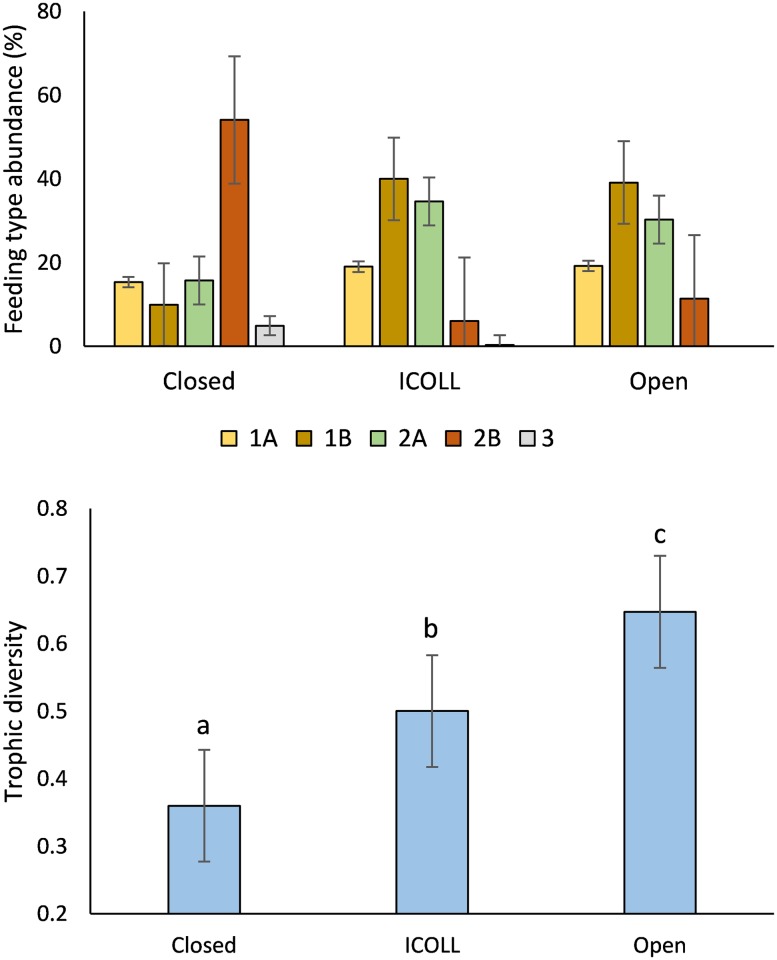
Relative abundance of nematode feeding types and index of trophic diversity (mean ±SE) in closed, intermittently open/closed (ICOLL) and open lagoons. Different letters indicate significant differences (p<0.05) among lagoon typology. (1A) selective deposit feeders, (1B) nonselective deposit feeders, (2A) epigrowth feeders, (2B) predators/omnivores and (3) vascular plant feeders.

Analysis performed on Euclidian distance matrices. P(MC): p-value obtained with Monte Carlo permutation test. Bold values indicate significant differences at p<0.05. For the results of pair-wise tests, see Figs 3 and 4, and S3, S4 and S5 Tables.

Nematode trophic structure differed significantly among lagoon typologies. While closed lagoons were largely dominated by predator/omnivores (mean of 54%), in ICOLLs and open lagoons nonselective deposit feeders and epigrowth feeders were significantly more abundant (mean of 39% and 32% respectively) (Fig 4 and S5 Table). Selective deposit feeders (with a mean around 16%) did not differ significantly among typologies (S5 Table). Abundances of vascular plant feeders significantly decreased with openness (Fig 4, S5 and S6 Tables). The index of trophic diversity was significantly lower in closed lagoons, intermediate in ICOLLs and higher in the open lagoons (Fig 4 and Table 2). Significant variations in trophic diversity among lagoons within individual typology and at scale of locations within the lagoon were not detected (Table 2).

### Connectivity and assemblage similarities

**Fig 5:**
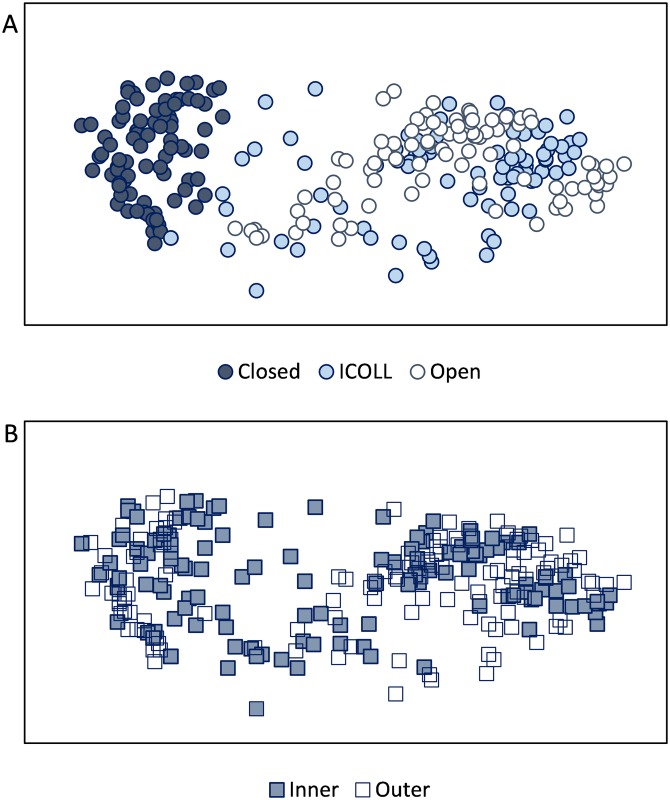
nMDS ordinations for log-transformed nematode abundances. (A) closed, intermittently open/closed (ICOLL) and open lagoons. (B) inner and outer portions of coastal lagoons. Stress 0.16.

The non-metric multidimensional scaling (nMDS) analysis revealed substantial differences in nematode assemblages between connected and closed lagoons, but not between locations within the lagoons (Fig 5). The results of the PERMANOVA showed that the greatest variation in the data dissimilarities occurred due to differences in connectivity rather than between locations within lagoons (Table 3). The statistical tests confirmed that nematode assemblages of closed lagoons differed significantly from more connected ones (Table 3). The PERMANOVA tests also revealed that nematode assemblages of inner and outer portion of closed lagoons did not differ significantly, whilst in the ICOLLS and open they did (Table 3). The analysis of the average similarity between/within lagoons showed that nematodes assemblages from the open lagoons were more similar to each other than those from the closed ones (Table 3). As lagoons lose connectivity with the sea, nematode composition became more dissimilar. Internal similarities, conversely, were higher within closed lagoons, decreasing as lagoons gain connectivity. The results of these analyses were consistent whether analysed by means of presence/absence or relative abundances.

**Table 3:** Permutational analysis of variance (PERMANOVA) results testing effects of typology (Closed, ICOLL and Open), lagoon (15 lagoons) and sampling location (inner and outer) on nematode assemblages.

Sources of variation	df	SS	MS	Pseudo-F	P(MC)
Typology	2	225730	112860	5.9	**0.001**
Lagoon (Typology)	12	230050	19171	2.43	**0.001**
Location[Lagoon(Typology)]	15	117950	7863	8.26	**0.001**
Residual	235	223700	951		
Pair-wise tests					
Lagoons compared	P(MC)	Inner vs outer within lagoons			P(MC)
Open x ICCOL	0.219	Open			**0.03**
Open x Close	**0.001**	ICCOL			**0.001**
ICCOL x Close	**0.001**	Close			0.076
	Average similarity between and within lagoons				
	Between lagoons				
		Open		ICOLL	Closed
	Open	36.166		-	-
	ICOLL	30.589		44.566	-
	Closed	12.009		16.315	51.256
	Average similarity within lagoons				
		Open		ICOLL	Closed
	Inner x outer	30.166		43.951	50.402
	Inner x inner	40.115		47.118	50.980
	Outer x outer	38.115		49.204	54,53

Analyses performed on Bray–Curtis dissimilarities of fourth root transformed nematode abundances. P(MC): p-value obtained with Monte Carlo permutation test. Bold values indicate significant differences at p<0.05.

**Fig 6:**
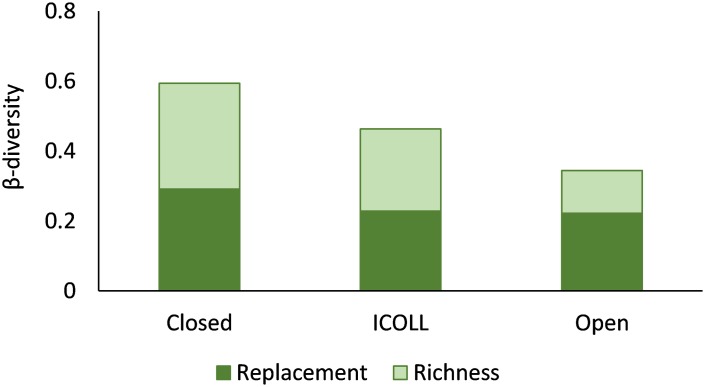
Nematode variation in genera composition (total mean β-diversity), contribution of replacement and richness differences (gray dots) and within in inner outer portions of lagoons (columns). Different letters indicate significant differences (p<0.05) among lagoon types.

The variability in genera composition (β-diversity) differed significantly according to lagoon typology and location (Table 4). Total β-diversity was significantly higher in closed lagoons, intermediate in ICOLLs and lower in the open lagoons (Fig 6 and S7 Table). Significant variations in β-diversity among lagoons within individual typology were mainly detected in closed ones (S8 Table). At the scale of locations within the lagoon, the genera variability was lower than at the scale of lagoon. Total genera variability between locations varied significantly in open lagoons and ICOLLs, but not in closed ones (S9 Table). The relative contributions of replacement and richness components to the nematode genera variability also differed significantly among lagoon typologies, with and increasing dominance of replacement over richness as lagoon connectivity increased (Fig 6).

**Table 4:** Permutational analysis of variance (PERMANOVA) results of the effects of lagoon typology (Open, ICCOL and Closed), lagoons (5 open, 5 ICOLL and 5 closed) and location (inner and outer) on the total β-diversity and decomposed replacement and richness differences.

	Sources of variation	df	SS	MS	PseudoF	P(MC)
Total β-diversity	Typology	2	29.875	14.937	9.2767	**0.001**
	Lagoon (Typology)	12	19.445	1.6204	2.4662	**0.001**
	Location[Lagoon(Typology)]	15	9.8702	0.6580	6.567	**0.001**
	Residual	229	22.946	0.1002		
Species replacement	Typology	2	9.3098	4.6549	9.4516	**0.004**
	Lagoon (Typology)	12	5.9469	0.4955	2.7067	**0.0381**
	Location[Lagoon(Typology)]	15	2.7501	0.1833	4.7777	**0.001**
	Residual	229	8.7877	0.0383		
Species richness	Typology	2	29.875	14.937	9.2767	**0.001**
	Lagoon (Typology)	2	7.7552	3.8776	6.8406	**0.002**
	Location[Lagoon(Typology)]	12	6.8456	0.5704	2.7158	**0.01**
	Residual	15	3.1554	0.2103	6.4882	**0.001**

### Environmental variables and nematode assemblages

Salinity values increased with increasing connectivity and were significantly higher at the outer portions of ICOLLs and open lagoons; salinity did not vary within closed lagoons (S10, S11, S12 and S13 Tables). In general, granulometry was relatively homogeneous among the lagoons, with sediments composed of moderately sorted fine sands (mean grain size and sediment sorting did not vary significantly among nor within lagoons). Grain size, sand, silt + clay percentages did not vary significantly among typologies, nor among lagoons within individual typology (S10 Table). However, total organic content was higher in the inner portion of open lagoons and ICOLLs, while sand percentages were higher in the outer portion of more connected lagoons (S10 and S13 Tables).

**Fig 7:**
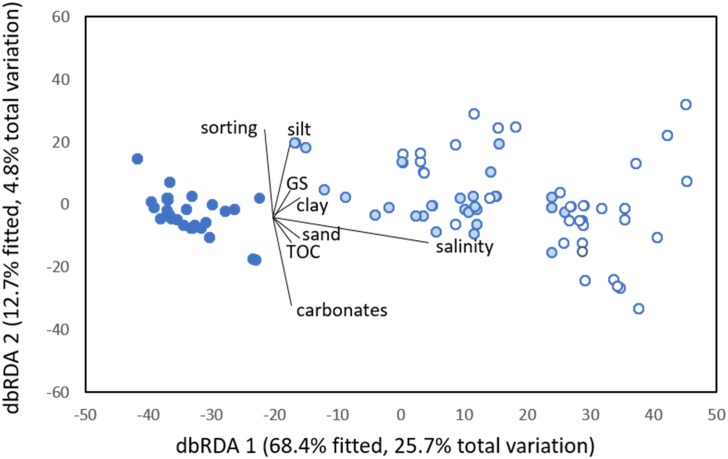
Distance-based RDA ordination relating environmental variables to the nematode composition. GS- grains size; TOC- total organic content; closed (dark blue circle), intermittently open/closed (light blue circle) and open (open circle).

The distance-based RDA ordination (Fig 7) indicated that the first two axes explained 30.5% of the variability in the faunal data and 81.1% of the relationship between nematode genera and the environmental variables (Fig 7). The first axis (responsible for 68.4% of the fitted model relating the fauna-environmental variables) was strongly related to salinity, and represented the connectivity gradient from the closed to the permanently open lagoons. The second axis, responsible for 12.7%, was related to sediment sorting, silt and carbonate percentages, and represented the variation within the lagoons.

## Discussion

Using particular lagoon status over space as replicates of their evolution over time, we observed that open and closed lagoons are mutually exclusive alternative states of equilibrium, and that ICOLLs are an intermediate or transition phase between them. The gradient in the structural connectivity between lagoons and the sea, due to their regime shifts, changes the movement of resources and consumers, and the internal physico-chemical gradients that directly affected the regional species diversity, abundance and trophic status. Whereas the lack of barriers and the fauna movements through the inlets increased similarity between the more connected lagoons, isolation increased variation in the composition of nematode assemblages with species losses and decrease of trophic diversity between closed lagoons.

Intermittently open/closed lagoons are particularly important in the understanding of biological and physico-chemical shifts between coastal lagoons/lakes. This is because, in the short-term, changes in the connectivity of ICOLLs leads to drastic environmental changes [9,10,11], shifting between the lacustrine or the lagoonar equilibrium state. The sampled ICOLLs in this study were not in the same status of closure (3 were closed and 2 were open) and our expectations were that nematode assemblages of the ICOLLs would be grouped with the closed or the open lagoon samples depending on their inlet state. Instead, we found that ICOLLs univariate and multivariate descriptors of the nematodes assemblages had an intermediate structure between lagoons and coastal lakes. ICOLLs are typically characterized by low freshwater inflow, which leads to sand berm formation across the mouth, preventing mixing with ocean water [37]. Besides, high intra and inter-annual variability in rainfalls and discharges are typical of ICOLLs [38]. A possible mechanistic explanation for the transitional structure of the nematode assemblages is the intermediate pattern of isolation compared to lagoons and coastal lakes. Increasing isolation from open ocean conditions also alters the structure of foraminiferans and macrobenthic assemblages, leading to a decrease in diversity and changes of species [22,39]. The higher diversity of nematode assemblages in brackish water compared to freshwater reflects both the input of marine species and the presence of strong environmental gradients and higher environmental heterogeneity. Overall freshwater nematode communities are impoverished when compared to marine and brackish systems [35]. Regarding nematode abundance, higher values in open lagoons and ICOLLs probably reflects the amount of organic matter. Although just marginally significant, closed lagoons had lower TOC than ICOLL and open ones. Moreover, TOC was significantly higher in the outer portion of open lagoons and ICOLLs, as observed for nematode abundances, giving support to the hypothesis that TOC plays a significant role in nematode abundances [40].

Our results further showed that similarities of the nematode assemblages within and between lagoons also change according to the stable state. While habitat connection and faunal exchange by open inlets increased similarity between more connected lagoons, with variations in the composition controlled by gradients, isolation increased variability of nematode assemblages between closed lagoons. At the same time, internal variability was higher within open lagoons than in closed lagoons, with ICOLL again assuming an intermediate position. This pattern may emerge as a result of the connectivity that modulates the degree to which the inlet state facilitates or impedes the exchange of matter, energy and specimens among landscape elements. Besides, differences in structural connectivity can lead to internal homogeneity or strong physico-chemical gradients that directly affect species composition.

While the low variability of nematode assemblages among lagoons is likely to be a result of faunal transport due to their physical link, the high dissimilarities of the assemblages between coastal lakes might be consequence of their spatially disconnection and exposure to different environmental conditions as a result of the discrete and variable surroundings. The coastal lakes could be colonized by different adjacent freshwater sources, by flooding events [41] or phoresy [42]. Moreover, some common taxa of freshwater and brackish habitats, such as enoplids and chromadorids, could be dispersed from the nearby coastal areas by wind, salt spray or sea foam [41]. Although nematode composition and abundance are known to be closed related to the lakes trophic state and related sediment characteristics [29,43,44], in the present study, all coastal lakes can be classified as oligotrophic, with bottoms composed of clean sandy sediments and very low total organic content (<0.5%). Our results indicated that the nematode assemblages of coastal lakes are primarily structured by the intrinsic properties within each lake and to a possible limited dispersion ability of nematodes between lakes.

Natural and gradual shifts from lagoons to lakes are long-term processes that result from large-scale (e.g. sea-level and climate changes) and local processes (e.g. sediment supply, alongshore drift, coastal morphology) [5,45]. The impoverishment of the nematode assemblages and the substitution of brackish water species by freshwater species also promotes a change in the trophic status of the benthic system and a significant decrease of trophic diversity. The dominant genera of closed lagoons *Semitobrilus* and *Trischistoma* are predators, while the genera *Desmodora* and *Theristus* are, respectively, epigrowth and non-selective deposit feeders [35,46]. These findings indicate that the availability of trophic resources is strongly affected by shifts from lagoons to lakes, resulting in loss of functional diversity. Similar result was also observed for meiofauna in hard-bottom macroalgal meadow/barren regime shift [47]. Besides, natural shifts may also interact with human interventions, increasing the speed of the shift and changing the dynamics of coastal lagoon evolution [48]. Our results from the large-scale sampling program showed that as lagoons lose connectivity, gradually shifting the state, local processes within individual ICOLLs and particularly within lakes become increasingly more important factors in the structuring of these communities than differences in large scale process (such as geomorphology or biogeography). The main implication of these findings is that depending on the local stable state we may end up with alternative regional pattern of biodiversity.

These findings also have direct implications for management and conservation plans of lagoon environments. As an intermediate state, ICOLLs would play a key role in the management of regime shifts and, based on our results, the most suitable approach for management purposes would be to consider each ICOLL as a unique situation requiring a localized approach, slowing environmental change towards the tipping point (e.g., sediment infill). In the particular case of subtropical coastal lagoons, this imposes additional difficulties as they are mostly distributed among unplanned populated areas. As ICOLLs typically have small river catchments, it makes them sensitive to changing inflow conditions [49]. Poor occupation practices within lagoon floodplains can result in pressure for intervention—dredging and bulldozing to artificially breach or close the lagoon inlet, potentially reducing resilience. Monitoring, establishment of local estuarine management plan and permanent policy review would ensure that the most ecologically appropriate and cost effective options are being implemented at any given location [50].

## Conclusions

We conducted an extensive sampling program, using specific lagoon status over space as replicates of their evolution over time, and observed that open and closed lagoons are mutually exclusive alternative states of equilibrium, and ICOLLs are an intermediate or transition phase between them. The gradual regime shift of coastal lagoons, as they lose connectivity with the sea, changes the movement of resources and consumers, and the internal physico-chemical gradients that directly affected regional diversity, abundance and trophic status. Absence of barriers increased the diversity of nematode assemblages and the similarity between the fauna of more connected lagoons. Isolation increased the variation in species composition between lagoons and similarities within lagoons. As local processes within individual lagoons become increasingly more important as they lose connectivity, depending on the local stable state an alternative regional pattern of biodiversity may emerge.

## Supporting information

S1 Table Location of sampled lagoons along Santa Catarina State, South Brazil. (DOCX)

S2 Table Nematode genera, environment, mean density (inds.10cm^-2^) and feeding type of nematode genera along coastal lagoons of Santa Catarina, South Brazil. Feeding types: (1A) selective deposit feeders, (1B) nonselective deposit feeders, (2A) epigrowth feeders, (2B) predators/omnivores and (3) vascular plant feeders.
(DOCX)

S3 Table Results from pair-wise PERMANOVA tests on univariate nematode descriptor and nematode assemblages for lagoons (5 open, 5 ICOLL and 5 closed) nested in typology (open, ICOLL, closed). P(MC): p-value obtained with Monte Carlo permutation test.
(DOCX)

S4 Table Results from pair-wise PERMANOVA tests on univariate nematode descriptor and nematode assemblages for location (inner vs outer) nested in lagoon and typology. (DOCX)

S5 Table Results from PERMANOVA tests on nematode feeding types for lagoon typology (Open, ICCOL and Closed), lagoons (15 sample lagoons) sampling location (inner and outer). P(MC): p-value obtained with Monte Carlo permutation test. 1A- selective deposit feeders; 1B- nonselective deposit feeders; 2A- epigrowth feeders; 2B- predators/omnivores; 3- vascular plant feeders.
(DOCX)

S6 Table Results from pair-wise PERMANOVA tests on nematode feeding types for lagoons typology. p-value obtained with Monte Carlo permutation test. 1B- nonselective deposit feeders; 2A- epigrowth feeders; 2B- predators/omnivores; 3- vascular plant feeders.
(DOCX)

S7 Table Results from pair-wise PERMANOVA tests on total beta diversity, and decomposed replacement and richness differences for lagoons typology. Bold values indicate significant differences at p<0.05.
(DOCX)

S8 Table Results from pair-wise PERMANOVA tests on total beta diversity, and decomposed replacement and richness differences for lagoons (5 open, 5 ICOLL and 5 closed) nested in typology (open, ICOLL, closed). P(MC): p-value obtained with Monte Carlo permutation test.
(DOCX)

S9 Table Results from pair-wise PERMANOVA tests total beta diversity, and decomposed replacement and richness differences for location (inner vs outer) nested in lagoon and typology. (DOCX)

S10 Table Results from PERMANOVA tests on environmental variables for lagoon typology (Open, ICCOL and Closed), lagoons (15 sample lagoons) sampling location (inner and outer). P(MC): p-value obtained with Monte Carlo permutation test.
(DOCX)

S11 Table Results from pair-wise PERMANOVA tests on environmental variables for lagoons typology. p-value obtained with Monte Carlo permutation test.
(DOCX)

S12 Table Results from pair-wise PERMANOVA tests on environmental variables for lagoons (5 open, 5 ICOLL and 5 closed) nested in typology (open, ICOLL, closed). P(MC): p-value obtained with Monte Carlo permutation test.
(DOCX)

S13 Table Results from pair-wise PERMANOVA tests on environmental variables for location (inner vs outer) nested in lagoon and typology. (DOCX)
