# Supplementary material for: Regime shifts in coastal lagoons: Evidence from free-living marine nematodes
Source: PLoS One. 2017 Feb 24;12(2):e0172366. doi: 10.1371/journal.pone.0172366 (PMC5325531; doi:10.1371/journal.pone.0172366)
Supplement: S1 Table — (DOCX) [file pone.0172366.s001.docx]

S1 Table. Location of sampled lagoons along Santa Catarina State, South Brazil.

| Name | Type | Latitude | Longitude |
| --- | --- | --- | --- |
| Peri | Closed | 27°43'53" | 48°30'45" |
| Caverá | Closed | 28°50'31.21" | 49°15'43.90" |
| Faxinal | Closed | 29°14'12.75" | 49°39'14.31" |
| Esteves | Closed | 28°41'13.33" | 49°01'35.37" |
| Jaguaruna | Closed | 27°46'21.81" | 48°29'13.98" |
| Lagoinha do Leste | ICOLL | 28°04'07.65" | 48°38'41.85" |
| Garopaba | ICOLL | 28°08'33.51" | 48°39'38.78" |
| Ibiraquera | ICOLL | 29°11'18.60" | 49°41'34.58" |
| Sombrio | ICOLL | 28°47'30.24" | 49°12'08.83" |
| Urusanga Velha | ICOLL | 27°32'31" | 48°26'59" |
| Conceição | Open | 28°22'45.30" | 48°47'50.98" |
| Laguna | Open | 28°36'12.75" | 48°53'19.64" |
| Camacho | Open | 26°13'57.70" | 48°30'38.65" |
| Barra Velha | Open | 26°35'52.20" | 48°39'24.14" |
| São Francisco | Open | 26°35'52.20" | 48°39'24.14" |
