# Supplementary material for: Regime shifts in coastal lagoons: Evidence from free-living marine nematodes
Source: PLoS One. 2017 Feb 24;12(2):e0172366. doi: 10.1371/journal.pone.0172366 (PMC5325531; doi:10.1371/journal.pone.0172366)
Supplement: S2 Table — Feeding types: (1A) selective deposit feeders, (1B) nonselective deposit feeders, (2A) epigrowth feeders, (2B) predators/omnivores and (3) vascular plant feeders. (DOCX) [file pone.0172366.s002.docx]

S2 Table. Nematode genera, environment, mean density (inds.10cm^-2^) and feeding type of nematode genera along coastal lagoons of Santa Catarina, South Brazil. Feeding types: (1A) selective deposit feeders, (1B) nonselective deposit feeders, (2A) epigrowth feeders, (2B) predators/omnivores and (3) vascular plant feeders.

| **Genus** | **Environment** | **Closed** | **ICOLL** | **Open lagoons** | **Feeding type** |
| --- | --- | --- | --- | --- | --- |
| *Acanthonchus* | Brackish | 0.04 | 0.00 | 0.50 | 2A |
| *Adoncholaimus* | Brackish | 0.00 | 1.27 | 0.65 | 2B |
| *Anoplostoma* | Brackish | 0.07 | 51.11 | 44.27 | 1B |
| *Anticoma* | Brackish | 0.00 | 0.18 | 0.26 | 1A |
| *Antomicron* | Brackish | 0.00 | 0.00 | 0.21 | 1A |
| *Austronema* | Brackish | 0.00 | 0.00 | 0.04 | ? |
| *Axonolaimus* | Brackish | 0.00 | 0.04 | 0.00 | 1B |
| *Bathylaimus* | Brackish | 0.00 | 0.00 | 0.04 | 1B |
| *Cheironchus* | Brackish | 0.00 | 0.22 | 0.00 | 2B |
| *Chromadorina* | Brackish | 0.00 | 31.80 | 12.21 | 2A |
| *Chromadorita* | Brackish | 0.00 | 0.48 | 0.49 | 2A |
| *Cobbia* | Brackish | 0.00 | 0.78 | 2.15 | 2A |
| *Comesoma* | Brackish | 0.00 | 0.00 | 1.65 | 1B |
| *Cricolaimus* | Brackish | 0.00 | 0.00 | 0.04 | 2A |
| *Cyatholaimus* | Brackish | 0.00 | 0.00 | 0.07 | 2A |
| *Dasynemoides* | Brackish | 0.00 | 0.00 | 0.25 | 1A |
| *Desmodora* | Brackish | 0.04 | 71.09 | 37.18 | 2A |
| *Dichromodora* | Brackish | 4.78 | 5.21 | 17.70 | 2A |
| *Doliolaimus* | Brackish | 0.00 | 0.00 | 0.07 | 2B |
| *Dorylaimus* | Brackish | 0.00 | 3.45 | 0.14 | 2A |
| *Epacanthion* | Brackish | 0.00 | 9.29 | 0.36 | 2B |
| *Epsilonema* | Brackish | 0.00 | 0.07 | 76.18 | 1A |
| *Fenestrolaimus* | Brackish | 0.00 | 0.56 | 0.00 | 1A |
| *Gammanema* | Brackish | 0.00 | 0.15 | 0.11 | 2B |
| *Graphonema* | Brackish | 0.00 | 0.15 | 0.00 | 2A |
| *Halalaimus* | Brackish | 0.00 | 9.01 | 0.29 | 1A |
| *Halichoanolaimus* | Brackish | 0.00 | 1.26 | 1.47 | 2B |
| *Haliplectus* | Brackish | 0.00 | 0.00 | 0.04 | 1A |
| *Hopperia* | Brackish | 0.00 | 0.00 | 0.17 | 2B |
| *Hypodontolaimus* | Brackish | 0.57 | 0.06 | 0.07 | 2A |
| *Leptolaimus* | Brackish | 0.00 | 8.09 | 15.39 | 1A |
| *Linhomoeus* | Brackish | 0.00 | 0.37 | 5.62 | 2A |
| *Mesacanthion* | Brackish | 0.00 | 0.00 | 0.39 | 2B |
| *Metachromadora* | Brackish | 0.00 | 0.00 | 0.07 | 2A |
| *Metadesmolaimus* | Brackish | 0.00 | 0.26 | 0.07 | 1B |
| *Metalinhomoeus* | Brackish | 0.00 | 4.79 | 8.06 | 1B |
| *Metoncholaimus* | Brackish | 0.00 | 3.97 | 2.71 | 2B |
| *Microlaimus* | Brackish | 0.00 | 138.10 | 79.72 | 1A |
| *Minolaimus* | Brackish | 0.00 | 0.00 | 0.04 | 1A |
| *Molgolaimus* | Brackish | 0.00 | 18.30 | 1.90 | 1A |
| *Neotonchus* | Brackish | 0.00 | 0.04 | 0.47 | 2A |
| *Odontophora* | Brackish | 0.00 | 0.04 | 1.75 | 1B |
| *Paracanthonchus* | Brackish | 0.00 | 30.64 | 10.05 | 2A |
| *Paramicrolaimus* | Brackish | 0.00 | 0.00 | 0.14 | 2A |
| *Pomponema* | Brackish | 0.00 | 0.00 | 4.58 | 2B |
| *Pontonema* | Brackish | 0.00 | 0.00 | 0.04 | 2B |
| *Promonhystera* | Brackish | 0.00 | 0.00 | 9.63 | 1B |
| *Sabatieria* | Brackish | 0.00 | 3.10 | 3.40 | 1B |
| *Sphaerolaimus* | Brackish | 0.00 | 0.12 | 0.00 | 2B |
| *Spirina* | Brackish | 0.00 | 104.24 | 185.21 | 2A |
| *Spirobolbolaimus* | Brackish | 0.00 | 0.00 | 0.04 | 1B |
| *Synonchiella* | Brackish | 0.00 | 0.78 | 1.36 | 2B |
| *Syringolaimus* | Brackish | 0.00 | 0.17 | 0.12 | 2B |
| *Terschellingia* | Brackish | 0.00 | 8.12 | 8.46 | 1A |
| *Thalassomonhystera* | Brackish | 0.00 | 0.00 | 0.04 | 1B |
| *Trefusia* | Brackish | 0.00 | 0.04 | 7.16 | 1A |
| *Trichotheristus* | Brackish | 0.00 | 0.08 | 0.04 | 1B |
| *Wieseria* | Brackish | 0.00 | 0.04 | 0.00 | 1A |
| *Xyala* | Brackish | 0.00 | 0.04 | 0.00 | 1B |
| *Anonchus* | Freshwater/ Brackish | 4.25 | 8.82 | 14.86 | 1B |
| *Aphanolaimus* | Freshwater/ Brackish | 4.71 | 0.00 | 0.00 | 1A |
| *Aphanonchus* | Freshwater/ Brackish | 0.04 | 0.00 | 0.00 | 1A |
| *Chronogaster* | Freshwater/ Brackish | 0.00 | 0.07 | 0.04 | 1A |
| *Daptonema* | Freshwater/ Brackish | 0.00 | 32.09 | 10.93 | 1B |
| *Desmoscolex* | Freshwater/ Brackish | 0.00 | 1.96 | 0.32 | 1A |
| *Diplolaimella* | Freshwater/ Brackish | 0.00 | 0.85 | 0.11 | 1B |
| *Ethmolaimus* | Freshwater/ Brackish | 0.00 | 0.00 | 0.04 | 2A |
| *Ironus* | Freshwater/ Brackish | 5.10 | 0.04 | 0.00 | 2B |
| *Monhystrella* | Freshwater/ Brackish | 0.00 | 12.18 | 0.04 | 1B |
| *Mononchida* | Freshwater/ Brackish | 0.18 | 0.00 | 0.00 | 2B |
| *Monhystera* | Freshwater/ Brackish | 0.04 | 0.00 | 0.00 | 2B |
| *Neochromadora* | Freshwater/ Brackish | 0.00 | 17.33 | 7.01 | 2A |
| *Oncholaimus* | Freshwater/ Brackish | 0.00 | 9.79 | 7.51 | 2B |
| *Oxystomina* | Freshwater/ Brackish | 0.00 | 5.75 | 4.79 | 1A |
| *Paracyatholaimus* | Freshwater/ Brackish | 0.14 | 0.00 | 0.00 | 2A |
| *Paraplectonema* | Freshwater/ Brackish | 0.00 | 0.07 | 0.00 | 1A |
| *Parodontophora* | Freshwater/ Brackish | 0.00 | 10.93 | 17.89 | 1B |
| *Plectus* | Freshwater/ Brackish | 0.00 | 3.16 | 0.00 | 1A |
| *Polygastrophora* | Freshwater/ Brackish | 0.00 | 1.67 | 1.47 | 2B |
| *Punctodora* | Freshwater/ Brackish | 3.29 | 0.00 | 0.00 | 2A |
| *Rhabditis* | Freshwater/ Brackish | 0.00 | 0.04 | 0.00 | 1B |
| *Rhabdolaimus* | Freshwater/ Brackish | 0.00 | 5.80 | 0.00 | 1A |
| *Theristus* | Freshwater/ Brackish | 0.00 | 32.17 | 38.25 | 1B |
| *Viscosia* | Freshwater/ Brackish | 0.00 | 2.90 | 28.10 | 2B |
| *Westindicus* | Freshwater/ Brackish | 0.00 | 0.00 | 0.09 | 2B |
| *Afractinolaimus* | Freshwater | 0.21 | 0.00 | 0.00 | ? |
| *Afrodorylaimus* | Freshwater | 0.00 | 0.88 | 0.00 | 2B |
| *Alaimina* | Freshwater | 0.00 | 0.04 | 0.00 | 1A |
| *Calodorylaimus* | Freshwater | 0.00 | 0.04 | 0.00 | 3 |
| *Caviputa* | Freshwater | 0.00 | 0.04 | 0.00 | 1A |
| *Comiconchus* | Freshwater | 0.04 | 0.00 | 0.00 | 2B |
| *Cylindrolaimus* | Freshwater | 0.00 | 0.04 | 0.00 | 1A |
| *Desmolorenzenia* | Freshwater | 0.00 | 0.26 | 0.00 | 1A |
| *Hirschmanniella* | Freshwater | 0.00 | 0.15 | 0.00 | 3 |
| *Mesorhabditis* | Freshwater | 0.00 | 0.15 | 0.00 | 1B |
| *Mesotobrilus* | Freshwater | 0.00 | 0.15 | 0.00 | 2B |
| *Mononchoides* | Freshwater | 0.00 | 0.40 | 0.00 | 1B |
| *Neotobrilus* | Freshwater | 0.35 | 0.00 | 0.00 | 2B |
| *Paraphanolaimus* | Freshwater | 0.00 | 0.00 | 0.07 | 1A |
| *Prismatolaimus* | Freshwater | 0.00 | 0.04 | 0.00 | 1B |
| *Protorhabditis* | Freshwater | 0.00 | 0.04 | 0.00 | 1A |
| *Semitobrilus* | Freshwater | 12.17 | 0.00 | 0.00 | 2B |
| *Sinanema* | Freshwater | 0.00 | 0.56 | 0.00 | 1A |
| *Trachypleurosum* | Freshwater | 4.46 | 0.00 | 0.00 | 2B |
| *Trichistoma* | Freshwater | 13.59 | 0.00 | 0.00 | 2B |
| *Udonchus* | Freshwater | 0.00 | 0.04 | 0.00 | 1A |
