# Supplementary material for: Regime shifts in coastal lagoons: Evidence from free-living marine nematodes
Source: PLoS One. 2017 Feb 24;12(2):e0172366. doi: 10.1371/journal.pone.0172366 (PMC5325531; doi:10.1371/journal.pone.0172366)
Supplement: S3 Table — P(MC): p-value obtained with Monte Carlo permutation test. (DOCX) [file pone.0172366.s003.docx]

S3 Table. Results from pair-wise PERMANOVA tests on univariate nematode descriptor and nematode assemblages for lagoons (5 open, 5 ICOLL and 5 closed) nested in typology (open, ICOLL, closed). P(MC): p-value obtained with Monte Carlo permutation test.

|  |  | Richness | | Diversity | | Density | | Nematodes | |
| --- | --- | --- | --- | --- | --- | --- | --- | --- | --- |
| Typology | Lagoons compared | t | P(MC) | t | P(MC) | t | P(MC) | t | P(MC) |
| Open | Barra Velha, Camacho | 0.08 | 0.947 | 0.70 | 0.568 | 0.50 | 0.604 | 0.80 | 0.947 |
| Open | Barra Velha, S.F.Sul | 3.91 | 0.063 | 3.86 | 0.069 | 3.29 | 0.09 | 3.91 | 0.063 |
| Open | Barra Velha, Conceição | 2.55 | 0.124 | 0.81 | 0.493 | 3.02 | 0.103 | 2.55 | 0.124 |
| Open | Barra Velha, Laguna | 0.57 | 0.585 | 0.71 | 0.954 | 0.38 | 0.976 | 0.57 | 0.585 |
| Open | Camacho, S.F.Sul | 2.57 | 0.124 | 3.41 | 0.076 | 2.04 | 0.199 | 2.57 | 0.124 |
| Open | Camacho, Conceição | 2.04 | 0.197 | 1.09 | 0.397 | 2.94 | 0.088 | 2.04 | 0.197 |
| Open | Camacho, Laguna | 0.54 | 0.627 | 0.37 | 0.772 | 0.62 | 0.6 | 0.54 | 0.627 |
| Open | S.F.Sul, Conceição | 5.30 | 0.032 | 2.10 | 0.157 | 6.29 | 0.03 | 5.30 | 0.032 |
| Open | S.F.Sul, Laguna | 2.72 | 0.121 | 2.61 | 0.112 | 3.82 | 0.065 | 2.72 | 0.121 |
| Open | Conceição, Laguna | 1.17 | 0.332 | 0.61 | 0.618 | 3.66 | 0.065 | 1.17 | 0.332 |
| ICOLL | Garopaba, Sombrio | 1.13 | 0.375 | 1.43 | 0.293 | 0.89 | 0.458 | 1.13 | 0.375 |
| ICOLL | Garopaba, Urusanga | 0.92 | 0.47 | 0.78 | 0.525 | 1.16 | 0.368 | 0.92 | 0.47 |
| ICOLL | Garopaba, Ibiraquera | 1.08 | 0.401 | 0.77 | 0.503 | 0.39 | 0.745 | 1.08 | 0.401 |
| ICOLL | Garopaba, Lagoinha | 1.02 | 0.392 | 0.45 | 0.703 | 0.93 | 0.425 | 1.02 | 0.392 |
| ICOLL | Sombrio, Urusanga | 0.15 | 0.886 | 0.21 | 0.846 | 0.49 | 0.679 | 0.15 | 0.886 |
| ICOLL | Sombrio, Ibiraquera | 2.98 | 0.102 | 3.39 | 0.069 | 1.76 | 0.217 | 2.98 | 0.102 |
| ICOLL | Sombrio, Lagoinha | 2.80 | 0.111 | 2.11 | 0.169 | 2.14 | 0.171 | 2.80 | 0.111 |
| ICOLL | Urusanga, Ibiraquera | 1.83 | 0.201 | 1.15 | 0.365 | 1.73 | 0.211 | 1.83 | 0.201 |
| ICOLL | Urusanga, Lagoinha | 1.73 | 0.239 | 0.62 | 0.57 | 2.01 | 0.188 | 1.73 | 0.239 |
| ICOLL | Ibiraquera, Lagoinha | 1.18 | 0.125 | 2.38 | 0.143 | 1.02 | 0.432 | 1.13 | 0.865 |
| Closed | Peri, Jaguaruna | 0.96 | 0.46 | 1.92 | 0.2 | 0.42 | 0.715 | 0.96 | 0.46 |
| Closed | Peri, Faxinal | 0.16 | 0.882 | 0.13 | 0.914 | 0.52 | 0.633 | 0.16 | 0.882 |
| Closed | Peri, Laranjal | 3.13 | 0.117 | 3.83 | 0.062 | 2.25 | 0.158 | 3.13 | 0.117 |
| Closed | Peri, Tapera | 8.05 | 0.013 | 5.49 | 0.037 | 2.22 | 0.168 | 8.05 | 0.013 |
| Closed | Jaguaruna, Faxinal | 0.57 | 0.581 | 0.76 | 0.514 | 0.71 | 0.5 | 0.57 | 0.581 |
| Closed | Jaguaruna, Laranjal | 1.89 | 0.21 | 3.42 | 0.086 | 1.44 | 0.266 | 1.89 | 0.21 |
| Closed | Jaguaruna, Tapera | 1.12 | 0.36 | 1.15 | 0.368 | 0.72 | 0.546 | 1.12 | 0.36 |
| Closed | Faxinal, Laranjal | 1.03 | 0.389 | 0.86 | 0.447 | 1.56 | 0.279 | 1.03 | 0.389 |
| Closed | Faxinal, Tapera | 1.87 | 0.228 | 1.45 | 0.293 | 2.82 | 0.118 | 1.87 | 0.228 |
| Closed | Laranjal, Tapera | 22.1 | 0.005 | 8.69 | 0.018 | 1.44 | 0.266 | 22.1 | 0.005 |
