# Supplementary material for: Regime shifts in coastal lagoons: Evidence from free-living marine nematodes
Source: PLoS One. 2017 Feb 24;12(2):e0172366. doi: 10.1371/journal.pone.0172366 (PMC5325531; doi:10.1371/journal.pone.0172366)
Supplement: S4 Table — (DOCX) [file pone.0172366.s004.docx]

S4 Table. Results from pair-wise PERMANOVA tests on univariate nematode descriptor and nematode assemblages for location (inner vs outer) nested in lagoon and typology.

|  | |  | Richness | | | Diversity | | Density | | Nematodes | |
| --- | --- | --- | --- | --- | --- | --- | --- | --- | --- | --- | --- |
| Typology | | Lagoon | t | | P(MC) | t | P(MC) | t | P(MC) | t | P(MC) |
| Open | Barra Velha | | | 2.0595 | 0.052 | 2.2169 | 0.029 | 5.9554 | 0.001 | 2.0526 | 0.014 |
| Open | | Camacho | 3.449 | | 0.004 | 3.1294 | 0.01 | 8.3668 | 0.001 | 2.8372 | 0.001 |
| Open | | S.F.Sul | 2.4054 | | 0.021 | 2.5378 | 0.015 | 5.2025 | 0.001 | 2.2032 | 0.001 |
| Open | | Conceição | 2.2001 | | 0.052 | 1.6778 | 0.048 | 4.193 | 0.003 | 3.1334 | 0.001 |
| Open | | Laguna | 4.8023 | | 0.001 | 3.5429 | 0.004 | 3.4006 | 0.007 | 3.7321 | 0.001 |
| ICOLL | | Garopaba | 4.2108 | | 0.002 | 4.8428 | 0.001 | 1.9484 | 0.085 | 3.9103 | 0.001 |
| ICOLL | | Sombrio | 4.7169 | | 0.001 | 1.8514 | 0.093 | 3.313 | 0.005 | 4.5573 | 0.001 |
| ICOLL | | Urussanga | 7.079 | | 0.001 | 5.6055 | 0.001 | 2.3386 | 0.026 | 4.3905 | 0.001 |
| ICOLL | | Ibiraquera | 2.2563 | | 0.032 | 2.0458 | 0.07 | 4.277 | 0.001 | 2.5148 | 0.001 |
| ICOLL | | Lagoinha | 2.2425 | | 0.047 | 0.24102 | 0.807 | 2.2143 | 0.048 | 3.7002 | 0.001 |
| Closed | | Peri | 0.66462 | | 0.496 | 0.47063 | 0.649 | 1.2524 | 0.22 | 1.3067 | 0.179 |
| Closed | | Jaguaruna | 1.0332 | | 0.076 | 1.9918 | 0.063 | 5.0613 | 0.001 | 1.3662 | 0.182 |
| Closed | | Faxinal | 0.6463 | | 0.419 | 1.0023 | 0.076 | 1.6763 | 0.123 | 1.2682 | 0.211 |
| Closed | | Laranjal | 0.37872 | | 0.74 | 0.2897 | 0.777 | 0.35087 | 0.735 | 1.4595 | 0.086 |
| Closed | | Tapera | 0.15385 | | 0.878 | 1.9463 | 0.088 | 1.546 | 0.134 | 1.4265 | 0.085 |
