# Supplementary material for: Regime shifts in coastal lagoons: Evidence from free-living marine nematodes
Source: PLoS One. 2017 Feb 24;12(2):e0172366. doi: 10.1371/journal.pone.0172366 (PMC5325531; doi:10.1371/journal.pone.0172366)
Supplement: S5 Table — P(MC): p-value obtained with Monte Carlo permutation test. 1A- selective deposit feeders; 1B- nonselective deposit feeders; 2A- epigrowth feeders; 2B- predators/omnivores; 3- vascular plant feeders. (DOCX) [file pone.0172366.s005.docx]

S5 Table. Results from PERMANOVA tests on nematode feeding types for lagoon typology (Open, ICCOL and Closed), lagoons (15 sample lagoons) sampling location (inner and outer). P(MC): p-value obtained with Monte Carlo permutation test. 1A- selective deposit feeders; 1B- nonselective deposit feeders; 2A- epigrowth feeders; 2B- predators/omnivores; 3- vascular plant feeders.

| Variable | Source of variation | df | SS | MS | Pseudo-F | P(MC) |
| --- | --- | --- | --- | --- | --- | --- |
|  | Typology | 2 | 19.234 | 9.6172 | 1.743 | 0.243 |
| 1A | Lagoon (Typology) | 12 | 66.623 | 5.5519 | 1.723 | 0.185 |
|  | Location[Lagoon(Typology)] | 15 | 48.405 | 3.227 | 1.863 | 0.061 |
|  | Residual | 235 | 101.75 | 0.4443 |  |  |
|  | Typology | 2 | 49467 | 24733 | 7.8481 | 0.006 |
| 1B | Lagoon (Typology) | 12 | 38052 | 3171 | 2.8263 | 0.053 |
|  | Location[Lagoon(Typology)] | 15 | 16851 | 3123.4 | 2.0079 | 0.071 |
|  | Residual | 235 | 62625 | 273.47 |  |  |
|  | Typology | 2 | 18326 | 9162.9 | 5.5745 | 0.024 |
| 2A | Lagoon (Typology) | 12 | 19836 | 1653 | 1.2808 | 0.32 |
|  | Location[Lagoon(Typology)] | 15 | 19385 | 1292.3 | 1.7073 | 0.41 |
|  | Residual | 235 | 62868 | 274.53 |  |  |
|  | Typology | 2 | 1.1428E5 | 57142 | 41 | 0.001 |
| 2B | Lagoon (Typology) | 12 | 16814 | 1401.1 | 1.3544 | 0.279 |
|  | Location[Lagoon(Typology)] | 15 | 15537 | 1035.8 | 1.4967 | 0.301 |
|  | Residual | 235 | 67836 | 296.23 |  |  |
|  | Typology | 2 | 15.66 | 7.8299 | 7.9895 | 0.007 |
| 3 | Lagoon (Typology) | 12 | 11.82 | 0.9850 | 1.4535 | 0.226 |
|  | Location[Lagoon(Typology)] | 15 | 10.177 | 0.6784 | 1.8219 | 0.132 |
|  | Residual | 235 | 55.058 | 0.2404 |  |  |
