# Supplementary material for: Regime shifts in coastal lagoons: Evidence from free-living marine nematodes
Source: PLoS One. 2017 Feb 24;12(2):e0172366. doi: 10.1371/journal.pone.0172366 (PMC5325531; doi:10.1371/journal.pone.0172366)
Supplement: S6 Table — p-value obtained with Monte Carlo permutation test. 1B- nonselective deposit feeders; 2A- epigrowth feeders; 2B- predators/omnivores; 3- vascular plant feeders. (DOCX) [file pone.0172366.s006.docx]

S6 Table. Results from pair-wise PERMANOVA tests on nematode feeding types for lagoons typology. p-value obtained with Monte Carlo permutation test. 1B- nonselective deposit feeders; 2A- epigrowth feeders; 2B- predators/omnivores; 3- vascular plant feeders.

|  | 1B | | 2A | | 2B | | 3 | |
| --- | --- | --- | --- | --- | --- | --- | --- | --- |
| Typology compared | t | P(MC) | t | P(MC) | t | P(MC) | t | P(MC) |
| Open, ICOLL | 0.2279 | 0.98 | 0.98761 | 0.365 | 1.5343 | 0.163 | 2.9828 | 0.034 |
| Open, closed | 3.8809 | 0.005 | 2.1329 | 0.037 | 6.6527 | 0.001 | 3.2302 | 0.015 |
| ICOLL, closed | 3.6955 | 0.008 | 3.278 | 0.007 | 6.7043 | 0.002 | 2.5795 | 0.041 |
