# Supplementary material for: Regime shifts in coastal lagoons: Evidence from free-living marine nematodes
Source: PLoS One. 2017 Feb 24;12(2):e0172366. doi: 10.1371/journal.pone.0172366 (PMC5325531; doi:10.1371/journal.pone.0172366)
Supplement: S7 Table — Bold values indicate significant differences at p<0.05. (DOCX) [file pone.0172366.s007.docx]

S7 Table. Results from pair-wise PERMANOVA tests on total beta diversity, and decomposed replacement and richness differences for lagoons typology. Bold values indicate significant differences at p<0.05.

|  | Total β-diversity | | Species replacement | | Species diversity | |
| --- | --- | --- | --- | --- | --- | --- |
| Typology compared | t | P(MC) | t | P(MC) | t | P(MC) |
| Open, ICOLL | 3.1284 | 0.001 | 1.412 | 0.2263 | 3.6859 | 0.001 |
| Open, closed | 3.8274 | 0.001 | 3.8196 | 0.013 | 3.296 | 0.003 |
| ICOLL, closed | 3.6081 | 0.001 | 3.492 | 0.0122 | 0.3685 | 0.864 |
