# Supplementary material for: Regime shifts in coastal lagoons: Evidence from free-living marine nematodes
Source: PLoS One. 2017 Feb 24;12(2):e0172366. doi: 10.1371/journal.pone.0172366 (PMC5325531; doi:10.1371/journal.pone.0172366)
Supplement: S8 Table — P(MC): p-value obtained with Monte Carlo permutation test. (DOCX) [file pone.0172366.s008.docx]

S8 Table. Results from pair-wise PERMANOVA tests on total beta diversity, and decomposed replacement and richness differences for lagoons (5 open, 5 ICOLL and 5 closed) nested in typology (open, ICOLL, closed). P(MC): p-value obtained with Monte Carlo permutation test.

|  |  | Total β-diversity | | Species replacement | | Species diversity | |
| --- | --- | --- | --- | --- | --- | --- | --- |
| Typology | Lagoons compared | t | P(MC) | t | P(MC) | t | P(MC) |
| Open | Barra Velha, Camacho | 1.3363 | 0.224 | 1.8736 | 0.1266 | 0.21368 | 0.915 |
| Open | Barra Velha, S.F.Sul | 1.5394 | 0.119 | 1.5774 | 0.2043 | 3.2685 | 0.048 |
| Open | Barra Velha, Conceição | 3.0791 | 0.014 | 4.0022 | 0.032 | 2.5893 | 0.062 |
| Open | Barra Velha, Laguna | 1.9306 | 0.056 | 3.3878 | 0.0195 | 0.49293 | 0.751 |
| Open | Camacho, S.F.Sul | 1.2765 | 0.2593 | 0.8985 | 0.4085 | 2.6328 | 0.08 |
| Open | Camacho, Conceição | 2.2222 | 0.031 | 2.7787 | 0.0493 | 1.8897 | 0.168 |
| Open | Camacho, Laguna | 1.1535 | 0.311 | 1.8237 | 0.1273 | 0.53002 | 0.698 |
| Open | S.F.Sul, Conceição | 2.0894 | 0.027 | 1.2089 | 0.3231 | 4.4699 | 0.013 |
| Open | S.F.Sul, Laguna | 1.5801 | 0.1081 | 0.8885 | 0.4099 | 2.9239 | 0.053 |
| Open | Conceição, Laguna | 1.622 | 0.088 | 2.4219 | 0.0596 | 1.1151 | 0.375 |
| ICOLL | Garopaba, Sombrio | 1.0705 | 0.398 | 1.0181 | 0.4462 | 1.0207 | 0.431 |
| ICOLL | Garopaba, Urusanga | 1.1087 | 0.355 | 1.5774 | 0.2043 | 0.89978 | 0.488 |
| ICOLL | Garopaba, Ibiraquera | 1.6591 | 0.1031 | 2.3204 | 0.0664 | 1.0476 | 0.417 |
| ICOLL | Garopaba, Lagoinha | 1.4306 | 0.18 | 1.9332 | 0.1299 | 0.94807 | 0.482 |
| ICOLL | Sombrio, Urusanga | 1.1929 | 0.3 | 2.0332 | 0.1095 | 0.38782 | 0.848 |
| ICOLL | Sombrio, Ibiraquera | 1.9074 | 0.052 | 1.6331 | 0.1626 | 2.185 | 0.106 |
| ICOLL | Sombrio, Lagoinha | 1.5696 | 0.115 | 1.2089 | 0.3231 | 2.0152 | 0.148 |
| ICOLL | Urusanga, Ibiraquera | 1.5556 | 0.1542 | 2.2567 | 0.0947 | 1.5396 | 0.217 |
| ICOLL | Urusanga, Lagoinha | 1.3261 | 0.216 | 1.6597 | 0.1893 | 1.4097 | 0.267 |
| ICOLL | Ibiraquera, Lagoinha | 1.5756 | 0.123 | 2.1441 | 0.0907 | 0.13232 | 0.971 |
| Closed | Peri, Jaguaruna | 2.1229 | 0.0474 | 2.6775 | 0.0652 | 0.79323 | 0.568 |
| Closed | Peri, Faxinal | 2.2777 | 0.0412 | 4.8358 | 0.0114 | 0.19436 | 0.969 |
| Closed | Peri, Laranjal | 2.2253 | 0.0541 | 2.0242 | 0.0958 | 2.0278 | 0.154 |
| Closed | Peri, Tapera | 2.67 | 0.0291 | 1.2156 | 0.2827 | 13.095 | 0.001 |
| Closed | Jaguaruna, Faxinal | 0.9033 | 0.5076 | 3.1564 | 0.0422 | 0.66079 | 0.629 |
| Closed | Jaguaruna, Laranjal | 1.4054 | 0.2336 | 0.9854 | 0.4085 | 1.5974 | 0.193 |
| Closed | Jaguaruna, Tapera | 1.5365 | 0.1748 | 1.6095 | 0.176 | 1.1265 | 0.391 |
| Closed | Faxinal, Laranjal | 1.1894 | 0.3555 | 1.7652 | 0.1524 | 0.65439 | 0.66 |
| Closed | Faxinal, Tapera | 1.3942 | 0.2136 | 1.6331 | 0.1626 | 1.8401 | 0.14 |
| Closed | Laranjal, Tapera | 1.6911 | 0.1449 | 1.2089 | 0.3231 | 6.0816 | 0.007 |
