# Supplementary material for: Regime shifts in coastal lagoons: Evidence from free-living marine nematodes
Source: PLoS One. 2017 Feb 24;12(2):e0172366. doi: 10.1371/journal.pone.0172366 (PMC5325531; doi:10.1371/journal.pone.0172366)
Supplement: S9 Table — (DOCX) [file pone.0172366.s009.docx]

S9 Table. Results from pair-wise PERMANOVA tests total beta diversity, and decomposed replacement and richness differences for location (inner vs outer) nested in lagoon and typology.

|  |  | Total β-diversity | | Species replacement | | Species diversity | |
| --- | --- | --- | --- | --- | --- | --- | --- |
| Typology | Lagoon | t | P(MC) | t | P(MC) | t | P(MC) |
| Open | Barra Velha | 2.4504 | 0.007 | 2.6695 | 0.0232 | 2.0052 | 0.058 |
| Open | Camacho | 3.0532 | 0.001 | 3.0582 | 0.005 | 2.9858 | 0.01 |
| Open | S.F.Sul | 2.505 | 0.001 | 2.4422 | 0.0291 | 1.9793 | 0.056 |
| Open | Conceição | 2.8969 | 0.001 | 2.5294 | 0.009 | 1.9841 | 0.054 |
| Open | Laguna | 2.7596 | 0.001 | 1.6846 | 0.064 | 4.7316 | 0.002 |
| ICOLL | Garopaba | 3.2687 | 0.001 | 2.9938 | 0.005 | 3.4403 | 0.002 |
| ICOLL | Sombrio | 3.4343 | 0.001 | 2.5758 | 0.017 | 4.4661 | 0.001 |
| ICOLL | Urussanga | 4.9214 | 0.001 | 3.7297 | 0.0226 | 4.6376 | 0.001 |
| ICOLL | Ibiraquera | 2.1275 | 0.001 | 1.7132 | 0.1202 | 2.2337 | 0.04 |
| ICOLL | Lagoinha | 3.3642 | 0.001 | 3.6428 | 0.006 | 0.29384 | 0.885 |
| Closed | Peri | 1.1588 | 0.273 | 1.8704 | 0.1694 | 3.1056 | 0.005 |
| Closed | Jaguaruna | 1.1560 | 0.270 | 1.7808 | 0.1919 | 0.5649 | 0.079 |
| Closed | Faxinal | 1.2918 | 0.188 | Negative | - | 0.13342 | 0.949 |
| Closed | Laranjal | 1.3891 | 0.151 | 2.5899 | 0.0504 | 2.0052 | 0.058 |
| Closed | Tapera | 1.3450 | 0.171 | 1.7222 | 0.1146 | 2.9858 | 0.01 |
