# Supplementary material for: Regime shifts in coastal lagoons: Evidence from free-living marine nematodes
Source: PLoS One. 2017 Feb 24;12(2):e0172366. doi: 10.1371/journal.pone.0172366 (PMC5325531; doi:10.1371/journal.pone.0172366)
Supplement: S10 Table — P(MC): p-value obtained with Monte Carlo permutation test. (DOCX) [file pone.0172366.s010.docx]

S10 Table. Results from PERMANOVA tests on environmental variables for lagoon typology (Open, ICCOL and Closed), lagoons (15 sample lagoons) sampling location (inner and outer). P(MC): p-value obtained with Monte Carlo permutation test.

| Variable | Source of variation | df | SS | MS | Pseudo-F | P(MC) |
| --- | --- | --- | --- | --- | --- | --- |
|  | Typology | 2 | 3313.5 | 1656.7 | 7.37 | 0.001 |
| Salinity | Lagoon (Typology) | 12 | 2703.2 | 225.27 | 4.007 | 0.008 |
|  | Location[Lagoon(Typology)] | 15 | 843.67 | 56.245 | 15.147 | 0.001 |
|  | Residual | 59 | 219.08 | 3.7133 |  |  |
|  | Typology | 2 | 18.71 | 9.354 | 0.9656 | 0.393 |
| CaCO3 (%) | Lagoon (Typology) | 12 | 18.71 | 9.3549 | 0.9656 | 0.461 |
|  | Location[Lagoon(Typology)] | 15 | 2.2677 | 0.1511 | 0.126 | 0.997 |
|  | Residual | 59 | 70.689 | 1.1981 |  |  |
|  | Typology | 2 | 12.639 | 6.3197 | 3.4946 | 0.05 |
| TOC (%) | Lagoon (Typology) | 12 | 21.737 | 1.8114 | 0.89857 | 0.566 |
|  | Location[Lagoon(Typology)] | 15 | 30.251 | 2.0168 | 4.144 | 0.001 |
|  | Residual | 59 | 28.714 | 0.4866 |  |  |
|  | Typology | 2 | 0.17212 | 0.0860 | 1.8006 | 0.208 |
| grain size (φ) | Lagoon (Typology) | 12 | 0.57473 | 0.0478 | 1.5607 | 0.196 |
|  | Location[Lagoon(Typology)] | 15 | 0.46054 | 0.0380 | 8.29 | 0.001 |
|  | Residual | 59 | 0.21851 | 0.0037 |  |  |
|  | Typology | 2 | 2.5512 | 1.2756 | 2.2496 | 0.146 |
| Sorting(φ) | Lagoon (Typology) | 12 | 6.8192 | 0.5682 | 4.5695 | 0.006 |
|  | Location[Lagoon(Typology)] | 15 | 1.8662 | 0.1244 | 4.2309 | 0.001 |
|  | Residual | 59 | 1.735 | 0.0294 |  |  |
|  | Typology | 2 | 1802.2 | 901.12 | 0.9858 | 0.407 |
| Sand (%) | Lagoon (Typology) | 12 | 10994 | 916.14 | 0.87006 | 0.585 |
|  | Location[Lagoon(Typology)] | 15 | 15803 | 1053.5 | 63.612 | 0.001 |
|  | Residual | 59 | 977.16 | 16.562 |  |  |
|  | Typology | 2 | 2553.5 | 1276.8 | 1.4805 | 0.261 |
| Silt+Clay (%) | Lagoon (Typology) | 12 | 10372 | 864.34 | 0.86327 | 0.603 |
|  | Location[Lagoon(Typology)] | 15 | 15 | 15027 | 1001.8 | 0.001 |
|  | Residual | 59 | 281.46 | 4.7705 |  |  |
