# Supplementary material for: Regime shifts in coastal lagoons: Evidence from free-living marine nematodes
Source: PLoS One. 2017 Feb 24;12(2):e0172366. doi: 10.1371/journal.pone.0172366 (PMC5325531; doi:10.1371/journal.pone.0172366)
Supplement: S11 Table — p-value obtained with Monte Carlo permutation test. (DOCX) [file pone.0172366.s011.docx]

S11 Table. Results from pair-wise PERMANOVA tests on environmental variables for lagoons typology. p-value obtained with Monte Carlo permutation test.

|  | Salinity | | TOC | |
| --- | --- | --- | --- | --- |
| Typology compared | t | P(MC) | t | P(MC) |
| Open, ICOLL | 1.5469 | 0.178 | 1.1773 | 0.291 |
| Open, closed | 3.7764 | 0.006 | 2.6262 | 0.021 |
| ICOLL, closed | 2.7761 | 0.033 | 2.5199 | 0.026 |
