# Supplementary material for: Regime shifts in coastal lagoons: Evidence from free-living marine nematodes
Source: PLoS One. 2017 Feb 24;12(2):e0172366. doi: 10.1371/journal.pone.0172366 (PMC5325531; doi:10.1371/journal.pone.0172366)
Supplement: S12 Table — P(MC): p-value obtained with Monte Carlo permutation test. (DOCX) [file pone.0172366.s012.docx]

S12 Table. Results from pair-wise PERMANOVA tests on environmental variables for lagoons (5 open, 5 ICOLL and 5 closed) nested in typology (open, ICOLL, closed). P(MC): p-value obtained with Monte Carlo permutation test.

|  |  | Salinity | | Carbonates | | Sorting | | |
| --- | --- | --- | --- | --- | --- | --- | --- | --- |
| Typology | Lagoons compared | t | P(MC) | t | P(MC) | t | P(MC) |  |
| Open | Camacho, Conceição | 0.1099 | 0.922 | 33.361 | 0.001 | 0.588 | 0.602 |  |
| Open | Camacho, Barra Velha | 3.4398 | 0.078 | 3.3 | 0.098 | 6.1371 | 0.027 |  |
| Open | Camacho, Laguna | 1.4138 | 0.287 | 1.6095 | 0.243 | 0.582 | 0.953 |  |
| Open | Camacho, SF Sul | 1.402 | 0.298 | 3.3 | 0.088 | 6.1371 | 0.029 |  |
| Open | Conceição, Barra Velha | 16.871 | 0.008 | 51.414 | 0.002 | 4.8254 | 0.027 |  |
| Open | Conceição, Laguna | 1.9831 | 0.174 | 36.469 | 0.001 | 0.60523 | 0.612 |  |
| Open | Conceição, SF Sul | 1.9757 | 0.184 | 51.414 | 0.001 | 4.8254 | 0.039 |  |
| Open | Barra Velha, Laguna | 1.2152 | 0.347 | 1.2182 | 0.335 | 5.7941 | 0.024 |  |
| Open | Barra Velha, SF Sul | 1.2516 | 0.353 | 1.3472 | 0.342 | 0 | 1 |  |
| Open | Laguna, SF Sul | 0.0183 | 0.987 | 1.2182 | 0.339 | 5.7941 | 0.026 |  |
| ICOLL | Garopaba, Ibiraquera | 2.3242 | 0.139 | 1.0194 | 0.425 | 1.3297 | 0.322 |  |
| ICOLL | Garopaba, Lagoinha | 0.78192 | 0.512 | 1.0893 | 0.379 | 0.542 | 0.965 |  |
| ICOLL | Garopaba, Urussanga | 0.51515 | 0.645 | 1.3472 | 0.342 | 0.68398 | 0.551 |  |
| ICOLL | Garopaba, Sombrio | 1.3402 | 0.307 | 2.094 | 0.16 | 2.2874 | 0.153 |  |
| ICOLL | Ibiraquera, Lagoinha | 5.9472 | 0.03 | 0.495 | 0.971 | 0.77398 | 0.506 |  |
| ICOLL | Ibiraquera, Urussanga | 5.791 | 0.029 | 1.0194 | 0.424 | 0.83398 | 0.517 |  |
| ICOLL | Ibiraquera, Sombrio | 7.1253 | 0.013 | 0.89814 | 0.463 | 2.5634 | 0.118 |  |
| ICOLL | Lagoinha, Urussanga | 0.5297 | 0.641 | 1.0893 | 0.374 | 0.63121 | 0.589 |  |
| ICOLL | Lagoinha, Sombrio | 0.84309 | 0.484 | 0.95864 | 0.433 | 2.0069 | 0.184 |  |
| ICOLL | Urussanga, Sombrio | 1.4528 | 0.274 | 2.094 | 0.173 | 0.27916 | 0.811 |  |
| Closed | Peri, Esteves | Denominator is 0 | | Denominator is 0 | | 1.1943 | 0.359 |  |
| Closed | Peri, Cavera | Denominator is 0 | | Denominator is 0 | | 1.1465 | 0.342 |  |
| Closed | Peri, Faxinal | Denominator is 0 | | Denominator is 0 | | 0.4555 | 0.705 |  |
| Closed | Peri, Jaguaruna | Denominator is 0 | | Denominator is 0 | | 0.88446 | 0.45 |  |
| Closed | Esteves, Cavera | Denominator is 0 | | Denominator is 0 | | 0.59076 | 0.618 |  |
| Closed | Esteves, Faxinal | Denominator is 0 | | Denominator is 0 | | 6.6242 | 0.029 |  |
| Closed | Esteves, Jaguaruna | Denominator is 0 | | Denominator is 0 | | 3.4578 | 0.073 |  |
| Closed | Cavera, Faxinal | Denominator is 0 | | Denominator is 0 | | 10.112 | 0.015 |  |
| Closed | Cavera, Jaguaruna | Denominator is 0 | | Denominator is 0 | | 13.802 | 0.008 |  |
| Closed | Faxinal, Jaguaruna | Denominator is 0 | | Denominator is 0 | | 6.0454 | 0.035 |  |
