# Supplementary material for: Regime shifts in coastal lagoons: Evidence from free-living marine nematodes
Source: PLoS One. 2017 Feb 24;12(2):e0172366. doi: 10.1371/journal.pone.0172366 (PMC5325531; doi:10.1371/journal.pone.0172366)
Supplement: S13 Table — (DOCX) [file pone.0172366.s013.docx]

S13 Table. Results from pair-wise PERMANOVA tests on environmental variables for location (inner vs outer) nested in lagoon and typology.

|  |  | Salinity | | TOC (%) | | grain size (φ) | | | Sand (%) | | | Silt+Clay (%) | | |
| --- | --- | --- | --- | --- | --- | --- | --- | --- | --- | --- | --- | --- | --- | --- |
| Typology | Lagoon | t | P(MC) | t | P(MC) | t | P(MC) | t | | P(MC) | t | | P(MC) |  |
| Open | Camacho | 16.273 | 0.001 | 0.93 | 0.391 | 4.11 | 0.021 | 0.68 | | 0.516 | 0.75323 | | 0.486 |  |
| Open | Conceição | 16.306 | 0.001 | 0.35 | 0.713 | 2.67 | 0.07 | 1.0433 | | 0.371 | 0.53036 | | 0.629 |  |
| Open | Barra Velha | 16.806 | 0.001 | 1.65 | 0.172 | 3.006 | 0.038 | 1.1712 | | 0.308 | 1.8843 | | 0.135 |  |
| Open | Laguna | 14.538 | 0.001 | 2.75 | 0.043 | 5.25 | 0.007 | 1.975 | | 0.134 | 2.1139 | | 0.099 |  |
| Open | S.F.Sul | 13.846 | 0.001 | 1.65 | 0.176 | 1.98 | 0.119 | 1.1712 | | 0.295 | 1.8843 | | 0.147 |  |
| ICOLL | Garopaba | 4.718 | 0.012 | 0.34 | 0.757 | 7.99 | 0.002 | 5.0402 | | 0.009 | 5.1554 | | 0.006 |  |
| ICOLL | Ibiraquera | 5.862 | 0.010 | 0.34 | 0.762 | 0.76 | 0.949 | 0.68952 | | 0.516 | 0.65669 | | 0.552 |  |
| ICOLL | Lagoinha | 2.1404 | 0.105 | 0.68 | 0.57 | 2,34 | 0.096 | 1.0433 | | 0.371 | 1.5932 | | 0.214 |  |
| ICOLL | Urussanga | 2.1404 | 0.105 | 300.1 | 0.001 | 45.17 | 0.001 | 1.1712 | | 0.308 | 704.83 | | 0.001 |  |
| ICOLL | Sombrio | 3.36 | 0.001 | 1.83 | 0.132 | 0.55 | 0.61 | 1.4091 | | 0.216 | 1.2913 | | 0.27 |  |
| Closed | Peri | Denominator is 0 | | 2.87 | 0.046 | 1.98 | 0.104 | 1.6001 | | 0.209 | Denominator is 0 | | |  |
| Closed | Jaguaruna | Denominator is 0 | | 1.17 | 0.312 | 1.44 | 0.203 | 2.773 | | 0.001 | 1.2898 | | 0.252 |  |
| Closed | Faxinal | Denominator is 0 | | 0.73 | 0.483 | 0.17 | 0.874 | 1.4329 | | 0.215 | Denominator is 0 | | |  |
| Closed | Laranjal | Denominator is 0 | | 0.95 | 0.421 | 0.395 | 0.714 | 2.2696 | | 0.073 | 1 | | 0.362 |  |
| Closed | Tapera | Denominator is 0 | | 1.36 | 0.241 | 0.396 | 0.739 | 1.4091 | | 0.216 | 0.80093 | | 0.459 |  |
